# Radiofrequency Ablation as an Alternative and Novel Procedure in the Intraepithelial Neoplasia Management: A Systematic Scoping Review

**DOI:** 10.1002/hsr2.70938

**Published:** 2025-06-23

**Authors:** Hamid Reihani, Sepehr Ebrahimi‐Dehkordi, Reza Moshfeghinia, Amirhossein Ghiasi‐Nejad, Kiarash Noorizadeh, Reza Heidari‐Soureshjani

**Affiliations:** ^1^ Student Research Committee, School of Medicine Shiraz University of Medical Sciences Shiraz Iran; ^2^ Student Research Committee Shahrekord University of Medical Sciences Shahrekord Iran; ^3^ Students' Scientific Research Center Tehran University of Medical Sciences Tehran Iran

**Keywords:** Barrett's esophagus, carcinoma in situ, endoscopic mucosal resection, intraepithelial neoplasia, radiofrequency ablation

## Abstract

**Background and Aims:**

Radiofrequency ablation (RFA) has emerged as a promising minimally invasive technique for eliminating intraepithelial neoplasia (IEN). We reviewed current evidence investigating the role of RFA in managing different types of IEN.

**Methods:**

A systematic review was conducted to assess the current evidence for the use of RFA in managing various types of IEN. A three‐step search strategy was employed, involving keyword extraction, comprehensive database searching (PubMed, Scopus, Web of Science, Embase, and Google Scholar), and reference screening.

**Results:**

Thirty‐two articles were included in this review, and most of the literature reported the features of RFA in the esophagus‐related IENs (68.7%). RFA treatment was also found to effectively address gastric (9.3%), anal (12.5%), and cervical (9.3%) IENs. The general opinion on using RFA in Barrett's esophagus (BE) was its combination therapy with the endoscopic mucosal resection (EMR) methods, which suggested better outcomes than RFA/EMR monotherapy. RFA was not preferred treatment for high‐grade gastric dysplasia, but it was effective for the low‐grade type.

**Conclusion:**

As a minimally invasive, safe, and effective procedure, RFA holds promise for addressing lesions associated with BE, gastric, anal, and cervical IEN; However, the scope and gaps related to RFA therapy require to be explored precisely.

## 1 Introduction

Intraepithelial neoplasia (IEN) is a precancerous condition characterized by the abnormal growth of cells in the epithelium, which can progress to invasive cancer if left untreated [1]. It can occur in various cells, such as the prostate, cervix, and esophagus [2]. Early diagnosis and treatment of IEN are crucial to prevent the progression of invasive cancer; hence, various therapeutic modalities, including surgery, cryotherapy, and laser and radiofrequency ablation, have recently been introduced [3]. While different treatment options exist, radiofrequency ablation (RFA) has emerged as a promising noninvasive technique for removing IENs with low‐grade (LGD) and high‐grade (HGD) dysplasia [4]. RFA is a minimally invasive technique that uses high‐frequency electrical energy to heat and destroy abnormal tissue. The procedure involves inserting a thin probe into the affected area, delivering the energy to the target tissue [5]. RFA has also been successfully used to treat various malignancies, including liver, lung, and renal cancer. Its feasibility and effectiveness have led to its application in managing various IENs [6]. Technically, radiofrequency ablation can be performed under local anesthesia, making it safe and well‐tolerated by patients. Additionally, minimal associated complications, short recovery time, and effectiveness in treating recurrent or persistent IEN have been reported in many trials [7, 8, 9].

While RFA offers several advantages, challenges remain, such as determining the optimal RFA modality, energy dosage, and its applicability to IENs beyond the esophagus. Current IEN treatments often involve significant morbidity and prolonged recovery times, negatively impacting patients' quality of life [10]. Surgical interventions, in particular, can pose substantial risks for patients with comorbidities. Combination therapy, integrating endoscopic procedures with RFA, offers a promising approach to enhance both treatment efficacy and patient safety [11]. Therefore, this study reviewed current evidence investigating the role of RFA therapeutics and their clinical perspective on eliminating IENs and precancerous lesions.

## 2 Method and Material

### 2.1 Inclusion Criteria

We included all participants with different types of IEN in studies, regardless of age, sex, race, etc., who were treated by RFA or RFA plus other therapeutic procedures or ablation techniques. A preliminary search identified the types of IEN as gastrointestinal, anal, biliary, cervical, pancreatic, penile, prostatic, vaginal, and vulvar. There was no geographic restriction in the preliminary search. We also added all types of original research and different study designs.

### 2.2 Exclusion Criteria

Review studies, editorials, letters, book chapters, non‑English language articles, and conceptually unrelated papers were excluded from our research.

### 2.3 Search Strategy

This study used a three‐step search method by conducting a broad search in PubMed/Medline, Scopus, Embase, and Web of Science (WOS) databases. In addition, the Google Scholar database has been used to include grey Literature in the study, and the results of the first 10 pages are included in our study. Initially, the study keywords/Mesh terms were extracted with a preliminary search; then, a comprehensive search protocol was designed for this study. Furthermore, we reviewed the similar articles section and the reference list of these articles to make sure to include relevant articles in the initial search. Our search strategy in PubMed was as follows: ((((((“Radiofrequency Ablation”[MeSH Terms]) OR (“Radiofrequency Ablation”[Title/Abstract])) OR (“Radio‐Frequency Ablation”[Title/Abstract])) OR (RFA[Title/Abstract])) OR ((Radiofrequency[tiab] AND Ablation[tiab]))) OR ((Radio‐Frequency[tiab] AND Ablation[tiab]))) AND (((((((“intraepithelial neoplasia”[Title/Abstract]) OR (IEN[Title/Abstract])) OR (“Intraepithelial Neoplasm”[Title/Abstract])) OR (“Intraepithelial Neoplasms”[Title/Abstract])) OR ((intraepithelial[tiab] AND neoplasia[tiab]))) OR ((Intraepithelial[tiab] AND Neoplasm[tiab]))) OR ((Intraepithelial[tiab] AND Neoplasms[tiab]))).

### 2.4 Study Selection

**Figure 1:**
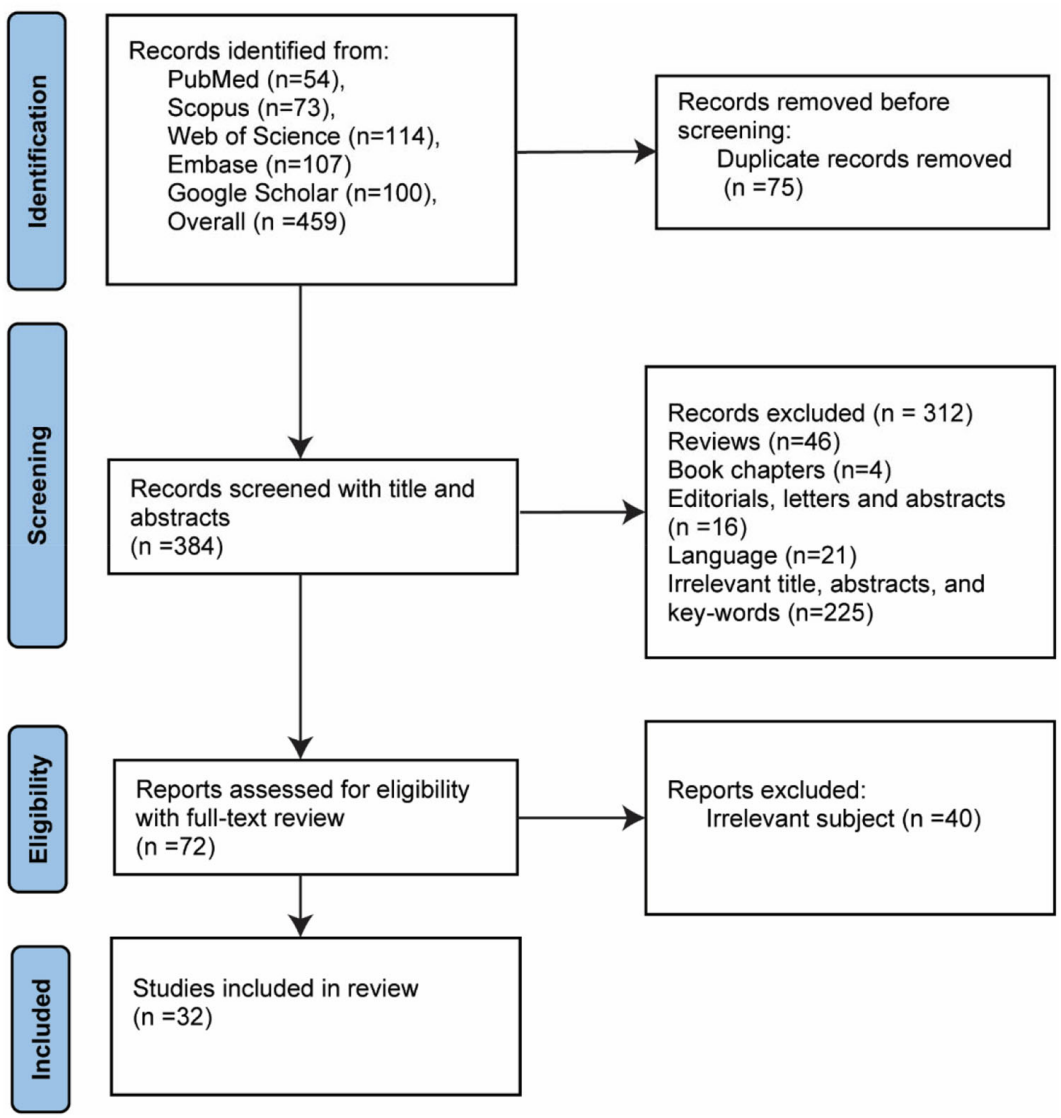
Data screening flowchart of our search strategy.

First, all selected studies were entered into EndNote, and the duplicates were removed. Afterward, R.M. and S.E. independently screened studies based on titles and abstracts (Figure 1). Then, the screened papers were selected by reviewing the full text. All possible disputes between the two reviewers were addressed and resolved by R.H.S.

### 2.5 Data Extraction

The data extraction for eligible articles was independently performed by two researchers (K.N. and A.G.) (Figure 1) based on the author's name, date of publication, study design, characteristics of the patient population (age, gender, sex ratio, etc.), specific aims, a summary of findings, and limitations.

### 2.6 Data Presentation

We presented the information in the form of a table and figures. Also, we review the potential gaps in the literature and, to some extent, provide suggestions for researchers interested in this field.

### 2.7 Methodological Framework

This scoping review was conducted following the rigorous methodological framework outlined by the Joanna Briggs Institute (JBI) for scoping reviews [12]. The JBI methodology provides a structured approach to identifying, charting, and synthesizing relevant literature while maintaining transparency and reproducibility.

### 2.8 Ethical Considerations

Regarding ethical considerations, as this study was a review of published literature and did not involve human participants, primary data collection, or direct patient involvement, ethical approval and informed consent were not applicable.

## 3 Result and Discussion

Thirty‐two articles were included in this review. There were 22 studies in the esophagus (70%), four anal (12%), three cervical (9%) and three gastric (9%).

### 3.1 RFA Procedures

RFA utilizes radiofrequency energy to eliminate dysplastic tissue. It can be applied circumferentially or focally to treat esophageal neoplasms [13]. RFA is administered using the BARRX system, which offers both circumferential (HALO 360) and focal (HALO 60, 90, Ultra, and through‐the‐scope) ablation devices [14, 15, 16]. Most studies employed a combination of circumferential and focal RFA. Standard circumferential ablation (1 × 12 J/cm^2^‐cleaning‐1 × 12 J/cm^2^) can be supplemented with focal ablation (2 × 15 J/cm^2^‐cleaning‐2 × 15 J/cm^2^) to improve outcomes. However, variations in treatment protocols, including the number of sessions and energy settings, exist across studies [17, 18, 19].

### 3.2 Esophageal IENs

This review includes 22 studies on esophageal IENs published between 2009 and 2022, consisting of six prospective studies (27.3%), three cohort studies (13.6%), two case series (9.1%), three randomized controlled trials (RCTs, 13.6%), seven retrospective studies (31.8%), and one decision analysis (4.5%), with a total sample size of 1413 participants, ranging from 1 to 176 participants per study.

#### 3.2.1 Remission, Success, or Recovery Rates

RFA has demonstrated remarkable efficacy in the treatment of Barrett's esophagus (BE) and early esophageal neoplasia. Across multiple studies, remission rates have been consistently high, ranging from 83% to 100%, depending on the patient population and treatment approach. In a study of 24 patients with BE ≤ 12 cm treated with RFA and endoscopic resection (ER), 95% neoplasia eradication increased to 100% after additional procedures. Intestinal metaplasia eradication was 88%, improving to 96% [20]. For BE ≥ 10 cm, RFA and ER achieved 83% remission of neoplasia and 79% intestinal metaplasia eradication, with no recurrence over 29 months [17]. For early esophageal squamous cell neoplasia (ESCN), RFA achieved 97% complete response at 12 months in one trial [21] and 86% sustained remission over 5 years, with 9% recurrence and 5% progression [14].

#### 3.2.2 RFA Alone, or in Combination With Endoscopic Resection?

The comparative efficacy of RFA alone versus RFA combined with endoscopic techniques has been extensively studied. Combining RFA with ER has shown superior outcomes compared to RFA alone. Combining EMR with RFA is more effective for treating Barrett's esophagus, offering better dysplasia eradication and manageable complication rates compared to RFA alone [22]. ESD of Barrett's neoplasia is feasible and safe, but does not achieve sufficient R0 resection rates to warrant its recommended use over piecemeal EMR. In combination with RFA, it can achieve complete eradication of neoplastic and non‐neoplastic Barrett epithelium [23]. Combining ER and RFA enhanced safety and efficacy in widespread BE [13].

#### 3.2.3 Side Effects: Types and Frequency

The safety profile of RFA has been thoroughly evaluated, revealing manageable adverse events. Strictures were among the most common complications, occurring in 14%–21% of cases, particularly following circumferential RFA [16, 21]. These strictures were effectively resolved through endoscopic dilation. Other complications included mucosal lacerations and intramucosal hematomas, particularly in patients with esophageal varices or ESCN [24]. Postablation lymphocytic esophagitis was another notable complication, with rates increasing from 7% pre‐ablation to 17% during the first surveillance and 43% at the final surveillance, demonstrating statistical significance [25]. Additionally, eosinophilic esophagitis occurred in 10% of RFA cases, significantly lower than the 27% observed with cryotherapy [26].

#### 3.2.4 Quality of Life (QOL) and Cost‐Effective Analysis

Quality of life (QOL) assessments have shown that patients treated with endoscopic therapy for early Barrett's neoplasia experience significant improvements compared to those undergoing surgical interventions [27]. Despite these improvements, psychological concerns such as anxiety about cancer recurrence persist. In one prospective study, patients who underwent endoscopic therapy reported high QOL scores but exhibited anxiety levels similar to those of patients treated surgically for advanced cancer, highlighting the need for psychological support in this population [27]. Cost‐effectiveness analyses have further supported the use of RFA and endoscopic therapy for early esophageal adenocarcinoma. A comparative study revealed that endoscopic therapy, consisting of endoscopic mucosal resection (EMR) followed by three RFA procedures, cost $17,000 and yielded 4.88 quality‐adjusted life years (QALYs). In contrast, esophagectomy cost $28,000 and yielded 4.59 QALYs. Esophagectomy became cost‐effective only if the risk of lymph node involvement exceeded 25%, which is uncommon in early‐stage cancers. These findings emphasize the economic and clinical benefits of endoscopic therapy, particularly for patients with a high surgical risk [28].

#### 3.2.5 Is It Suitable for Cirrhotic Patients With Esophageal Varices?

In patients with esophageal varices, the use of RFA has proven to be both effective and safe. A case series involving eight patients with varices and ESCN treated with circumferential RFA reported an initial complete response rate of 75%, which improved to 100% with additional focal RFA procedures within 12 months [24].

### 3.3 Gastric IENs

Gastric cancer is the fifth most common cancer and seventh in prevalence. In addition, gastric cancer is ranked third among the most lethal cancers in men after lung and liver cancers [29]. IEN in the stomach is associated with an increased risk of gastric cancer, so clinical concentrations are drawn to remove it. Similar to the esophagus, gastric IENs are classified as high‐grade and low‐grade. RFA application in gastric IENs was reported as 9.3% [29]. High‐grade IEN treatment is based on endotherapy and surgical procedures, whereas the low‐grade types would be managed by various techniques, including endoscopic submucosal dissection, photodynamic therapy, and RFA [29].

#### 3.3.1 RFA Versus Endotherapy in Low‐Grade Gastric IENs

Significant controversies exist around the factual effectiveness of the RFA or endoscopic techniques for low‐grade gastric IEN. Several studies have investigated endotherapy (EMR and dissection) to assess the safety and CR in low‐grade gastric IEN, which are high‐risk for complications and expensive [30]. These restrictions strengthen the idea of using radiofrequency ablation, as it is an affordable and straightforward method. Moreover, this procedure takes a short time (10–20 min), and the patient's recovery occurs faster than endoscopic procedures. RFA also does not require hospitalization and can be performed in outpatient settings [31].

#### 3.3.2 Efficacy and Potential of RFA in Gastric IENs

The first study to investigate the effect of RFA on gastric dysplasia was a case report by Baldaque‐Silva et al. They reported three cases that used the HALO ablation system with an interval of 8 weeks to eradicate gastric dysplasia. Following up at 2, 4, 6, 12, and 18 months later by endoscopic surveillance indicated no signs of dysplasia [32]. Leung et al. subsequently achieved similar findings in their clinical trial of four low‐grade gastric IEN patients. They observed the complete eradication of IEN from gastric tissue during a 1‐year follow‐up period [33]. Wang et al. retrospectively achieved the curative rate of 93.3% (in 3 months), 92.8% (in 6 months), 91.5% (in 1 year), 90.3% (in 2 years), 88.5% (in 3 years), 85.7% (in 4 years), and 83.3% (in 5 years). It was suggested that no significant association exists between age, sex, or lesion location and relapse chance. However, *H. pylori* infection and the disease course of > 1 year were reported as the main reasons for increasing the probability of relapse after RFA (Table 1) [31].

**Table 1:** Characteristics of the 32 included studies.

Author, year	Study type/participants (M/F)	Specific aims	Summary of findings	Limitations	Procedure/Frequency
Pohl et al.[28], 2009	Decision analysis 1 (1/0) Early BE carcinoma (65‐year‐old man)	BE: comparison of esophagectomy vs. endoscopic therapy (EMR plus RFA)	Endoscopic therapy for early BE adenocarcinoma is more effective and less expensive than esophagectomy.	Not investigated the long‐term outcomes	Prior EMR or ESD followed by three additional RFA procedures
Pouw et al. [20], 2010	Prospective 22 (NR) Early cancer, HGD	BE: comparison of post vs. pre‐RFA epithelium	Neither hidden glandular mucosa nor genetic abnormalities were detected histologically in the neo‐squamous epithelium (NSE) of post‐RFA. All patients' post‐RFA NSE revealed a normal distribution of stained nuclei in the epithelium of the basal layer. FISH analysis showed a normal diploid distribution in 100% of subjects. After a follow‐up time of 26 months, all patients had achieved permanent eradication of neoplasia and intestinal metaplasia (IM).	Immunohistochemistry is method‐dependent, so that the real mutation of P53 may be unrecognized. FISH has limitation to detect changes in BE epithelium, like P16 and P53 mutations.	Before EMR followed by circumferential/focal RFA (HALO 360 and HALO 90) At 2‐month intervals, until CR
Pouw et al. [20], 2010	Prospective 24 (20/4) Early cancer, HGD	BE: safety and efficacy of RFA plus baseline EMR	Early neoplasia in BE can be effectively and safely treated with RFA plus prior EMR of visible lesions. Neoplasia and IM were eradicated in 95% and 88% of patients, respectively. Complications were melena and dysphagia	Selection bias, because many EMR procedures were conducted before patient registration. Focal escape EMR in certain rare circumstances caused bias.	Before EMR or ESD followed by circumferential RFA (HALO 360, 12 J/cm^2^, 40 W/cm^2^)**/**Focal RFA (HALO 90, 15 J/cm^2^, 40 W/cm^2^) For IM: Focal RFA (HALO 90, 15 J/cm^2^, 2*2) At least 6 months after ER at 8‐week intervals, until CR
Alvarez Herrero et al. [17], 2011	Prospective 26 (21/5) Early cancer, HGD, LGD	BE: safety and efficacy of RFA with or without prior EMR	Low chance of recurrence during follow‐up. EMR for visible lesions, followed by RFA of residual BE, is a safe and effective treatment for neoplastic BE longer than 10 cm. CR‐neoplasia rate = 83%. CR‐IM rate = 79%. Complications were rare and included stenosis, mucosal laceration, hemorrhage needed transfusion, nausea, pain, etc. Ablations were unsatisfactory in 15% of patients because of poor healing and no regression, probably due to severe reflux disease.	Short follow up period	Before EMR and then **C**ircumferential RFA (HALO 360, 12 J/cm^2^, balloon‐based) /Focal RFA (HALO 90, 15 J/cm^2^, cap‐based) At least 6 months after EMR at 2 or 3‐month intervals, until CR
Galey et al. [34], 2011	Prospective 45 (43/2) Early cancer, HGD, LGD	ESCN: treatment and outcomes of multimodal endoscopic treatment	A minority of treated patients developed recurrent neoplasia, which is usually amenable to further endoscopic therapy. Complications are relatively minor and uncommon. In one patient, after three more EMRs and five RFA sessions, he has had eradication of metaplasia with no residual columnar line esophagus (CLE). Forty‐five patients treated for early esophageal neoplasia, endoscopic techniques achieved neoplasia eradication in 87.2% and intestinal metaplasia eradication in 56.4% over a 21.3‐month follow‐up. Metachronous neoplasia developed in 15.4% of patients, with three undergoing esophagectomy. No cases were unresectable or resulted in death.	Endoscopic treatment applicability to the majority of patients has not yet been established.	HGD: Cryoablation in two cycles of 20 s Adenocarcinoma: Cryoablation in three cycles of 20 s Visible lesion: EMR Visible non‐nodular lesions: RFA or cryoablation Ablation treatments repeated every 6–8 weeks
Bergman et al. [21], 2011	Prospective 29 (14/15) MGD, HGD, ESCC	ESCN: eradication by RFA	RFA was linked to the high rate of complete histological response (97%), no neoplastic progression, and an acceptable adverse event profile in patients with early ESCN (MGD, HGD, flat‐type ESCC). RFA may be helpful in the treatment of appropriately selected patients with early ESCN. EMR followed by RFA is safe and effective in studies of Barrett's neoplasia.	Single‐center study with a limited number of patients	A primary ablation (Within 3 months of the baseline endoscopy) by Circumferential RFA (HALO 360, 12 J/cm^2^, 40 W/cm^2^, balloon‐based) secondary ablation by Focal RFA (HALO 90, 12 J/cm^2^, 40 W/cm^2^) Permanent unstained lugol lesion (USLs) during follow‐up: three times in succession (12 J/cm^2^) with no cleaning
van Vilsteren et al. [18], 2011	Prospective case series 13 (8/5) HGD, ESCC	BE: effectiveness of RFA with and without EMR in BE	RFA with or without previous EMR for ESCC/HGD is possible and efficacious. Thirteen patients (10 with HGIN, 3 with ESCC), radiofrequency ablation (RFA) achieved complete histological response after a median of two sessions. Complications included two mucosal lacerations, one intramural hematoma, and three stenoses, with one perforation managed by stenting. No recurrences were noted over a 17‐month follow‐up.	The sample size was small. Because of expertise in the upper GI neoplasia field, the obtained result may be slightly difficult to translate for general gastroenterologists.	Before EMR and then primary ablation by circumferential RFA (HALO 360, 12 J/cm^2^, with cleaning phase, balloon‐based)**/**Focal RFA (HALO 90, 15 J/cm^2^, with cleaning phase) Endoscopic follow‐up in 2–3 months intervals
van Vilsteren et al. [19], 2011	RCT 47 (40/7) Early cancer, HGD	BE: comparison stepwise EMR vs. EMR plus RFA	CR neoplasia rate: SRER (100%) vs. EMR plus RFA (96%). CR‐IM rate: SRER (92%) vs. EMR plus RFA (96%). The stenosis confers significantly higher in SRER (88%) vs. EMR/RFA.	Patients treated at a referral center with extensive expertise in managing Barrett's neoplasia, so the results cannot be generalized to the other population.	Before EMR and then circumferential RFA (HALO 360, 12 J/cm^2^, 40 W/cm^2^, with cleaning phase)/focal RFA (HALO 90, 15 J/cm^2^, 40 W/cm^2^, with cleaning phase) at least 6–8 weeks after ER at 2 or 3‐month intervals, until CR
Caillol et al. [22], 2012	RCT 34 (28/6) HGD, LGD	BE: comparison of RFA plus EMR (group 1) vs. RFA without EMR (group 2)	CR in group 1 occurred in nine cases (56%) and PR in 100% of cases. CR in group 2 occurred in eight cases (44%) and PR in 14 cases (77%). The importance of obtaining histology before therapy should not be forgotten when using ablation.	Low number of RFA performed for patients due to high costs not covered by the health system.	Before EMR and then Circumferential RFA (HALO 360, 12 J/cm^2^, 40 W/cm^2^, balloon‐based) /focal RFA (HALO 90, 12 J/cm^2^, 40 W/cm^2^) EMR + RFA: 18 sessions RFA alone: 8 sessions
Neuhaus et al. [23], 2012	Prospective 30 (21/9) HGD, mucosal adenocarcinoma	BE: efficacy and safety of “waterjet‐assisted system ESD” (WESD) plus RFA	RFA combined with EMD can completely eradicate neoplastic and non‐neoplastic Barrett's epithelium. RFA of IM is advised for all CR neoplasia. WESD is safe and yields high rates of en bloc resection in selected patients with early Barrett's neoplasia. In 97% of the patients, a single session of WESD resulted in CR. WESD, like EMR, can be used in conjunction with RFA to achieve high rates of CR of IM and a low risk of neoplastic recurrence or metachronous lesions.	Limited number of patients. Uncontrolled single‐center study design. Not re‐evaluated the histology specimens of referring institutions.	Prior EMR or ESD and then HALO 360/HALO 90
Guarner‐Argente et al. [35], 2013	Retrospective 176 (145/31) HGD, IMC	BE: long‐term outcome in patients treated by endo‐luminal therapy (including RFA)	CR (for BE and HGD and/or intra‐mucosal carcinoma) with high success by EMR plus ablation techniques (RFA and others) Low risk of morbidity Endoscopic surveillance is needed after eradication. IM recurrence is detected in one‐third of successfully treated patients. The risk of recurrence increases when the lesion is multifocal dysplasia.	When a patient develops recurrence, they usually were returned for adjunctive therapy, and for this reason, the rate of IM recurrence in the long term may be underestimated. The retrospective nature of the study.	HALO 360 HALO 90 photodynamic therapy APC (60 W,1 L/min)
Halsey et al. [26], 2013	Retrospective 122 (96/26) Dysplastic BE or adenocarcinoma	BE: esophageal intra‐epithelial eosinophilic infiltration following endoscopic ablation	Eosinophilic infiltration is common in RFA therapy of BE, and it is associated with lesion length and treatment modality. The clinical significance of this eosinophilia is unclear. Eosinophilia may be a consequence of mucosal repetitive injury, food allergens, T helper response, and endoscopic intervention by triggering chronic inflammation. 10% of patients treated with RFA developed eosinophilic infiltration, which was less frequent than the 27% observed in patients treated with cryotherapy.	Information related to atopic/allergic history obtained only by post‐hoc interview.	—
Phoa et al. [36], 2013	Cohort study 55(45/10) HGD, early cancer	BE: 5‐year follow‐up of remission in patients who underwent RFA plus EMR (durability and efficacy)	An efficacy of 90% within 5‐year follow‐up. Three patients relapsed. The buried glands in endoscopic resection specimens were not found except for three subjects.	Patients were treated at a referral center with extensive expertise in managing Barrett's neoplasia, so the results cannot be generalized to other populations.	Before EMR and then circumferential RFA (HALO 360) and Focal RFA (HALO 90) at 3‐month intervals, until CR For Barrett's mucosa persisted after five ablations: escape endoscopic resection
Phoa et al. [37], 2014	RCT 136 (116/20) LGD	BE: whether endoscopic radiofrequency ablation could decrease the rate of neoplastic progression	Endoscopic ablative therapy is a superior management strategy for endoscopic surveillance in patients with BE and confirmed LGD. In patients with BE and a confirmed histological diagnosis of LGD, ablation substantially reduced the rate of neoplastic progression to HGD and adenocarcinoma. Radiofrequency ablation reduced the 3‐year progression risk of Barrett esophagus with low‐grade dysplasia to high‐grade dysplasia or adenocarcinoma by 25%	Patients treated at a referral center with extensive expertise in managing Barrett's neoplasia, so the results cannot be generalized to the other population.	Circumferential/focal RFA (HALO 360 and HALO 90) For residual persisted: a single session of EMR or argon plasma coagulation every 3 months, until CR
Bergman et al. [16], 2015	Prospective cohort 96 (52/44) HGD, MGD, ESCC	ESCN: the safety and effectiveness of RFA for early ESCN	In early ESCN patients, RFA is associated with a high rate of histological CR and a less adverse events profile. Focal RFA technique was standardized for therapy sessions and was well tolerated, allowing focal eradication of residual USLs.	Single‐center study	A primary ablation (Within 3 months of the baseline endoscopy) by circumferential RFA (HALO 360, 10‐12 J/cm^2^, 40 W/cm^2^, balloon‐based) secondary ablation by focal RFA (HALO 90, 12 J/cm^2^, 40 W/cm^2^)
Kissiedu et al. [25], 2016	Retrospective 102 (85/17) HGD, intramucosal carcinoma	BE: evaluate histological changes in esophageal postablation squamous mucosa compared with pre‐ablation	Lymphocytic esophagitis was associated with prior cryoablation, hyperlipidemia, and smoking history. It is essential for a pathologist to report the histologic findings and exclude other similar entities.	All of the patients in the study had an established diagnosis of gastroesophageal reflux disease. Improper patient allocation to specific therapy groups.	Circumferential (HALO 360) /focal RFA (HALO 90)/ cryotherapy (liquid nitrogen (−196°C) Ablation sessions repeated every 2–3 months, until complete absence of visible BE
Godat et al. [38], 2017	Retrospective 18 (17/1) HGD, superficial adenocarcinoma	Neoplastic BE: the efficiency of endotherapy in the case of neoplastic BE relapse	Endotherapy could be a treatment for neoplastic BE relapse.	The sample size was small. The results were from a single center. Most of the patients were men (17 of 18)	Prior EMR or ESD and then adjuvant Focal RF and APC depending on the lesion
Rosmolen et al. [27], 2017	Retrospective 126 (108/18) HGD, IM, early adenocarcinoma, advanced adenocarcinoma	Early Barrett's neoplasia: the overall quality of life and the fear of cancer recurrence	Endoscopic treatment of early esophageal cancer has a less negative impact on quality of life and symptoms than surgery. Despite better QOL scores, these patients exhibited significant anxiety about cancer recurrence, mirroring concerns seen in patients with advanced cancer treated surgically	Patients were not assigned randomly to the groups Short‐term follow‐up period (6 months).	—
Wang et al.[24], 2017	Case series 8 (8/0) HGD, LGD, early ESCN, intramucosal squamous carcinomas	ESCN in patients with esophageal varices: the efficacy and safety of RFA for early ESCNs in patients with well‐compensated cirrhosis accompanied by esophageal varices	RFA indicated desired treatment results, no neoplastic progression for treating early ESCNs in patients with compensated cirrhosis and esophageal varices. Adverse events included intramucosal hematomas and mucosal laceration, but no serious complications were noted	Low number of cases from a single institution.	Circumferential RFA (HALO 360, 12 J/cm^2^) for residual HGD: focal RFA (HALO 90, 12 J/cm^2^, 2–3 applications) for LGD: APC (1.5 L/min, 35 W) every 3 months, until CR
Krajciova et al. [15], 2019	Retrospective 136 (115/21) LGD, HGD, early adenocarcinoma, IM	BE: assess the long‐term outcomes of RFA in patients with BE	RFA is effective in achieving remission of BE‐related neoplasia. IM or neoplasia recurrence rates were low but not negligible. After a successful RFA for BE, the patients still need endoscopic surveillance. Cancer diagnosis was a risk factor for recurrent IM after RFA.	Not recognized the risk factors for relapse of the neoplasia	Prior EMR or ESD and then circumferential RFA (HALO 360, 12 J/cm^2^)/focal RFA (HALO 90, 12 J/cm^2^) every 2–3 months, until CR
Yu et al. [14], 2019	Cohort 78 (41/37) MGD, HGD, ESCC	ESCN: longer‐term outcomes after RFA for ESCN	The great majority (86%) of patients with ESCN or mucosal ESCC who had CR (no residual disease) within 12 months after initial RFA treatment, experienced sustained eradication of neoplasia during an additional four years of follow up. RFA may be best suited for the treatment of noninvasive epithelial neoplasia (i.e., MGD or HGD), and cannot be recommended for the treatment of ESCC.	Suboptimal follow‐up with protocol disregarding in a substantial number of patients The use of different RFA regimens at the baseline	Circumferential RFA (HALO 360)/focal RFA (HALO 90, 12 J/cm^2^) at 3‐month intervals, until CR
Godat et al.[12], 2022	Retrospective 89(79/10) HGD, IM, early adenocarcinoma	BE: complementary RFA after widespread EMR of neoplastic BE in daily practice	The combination of EMR and RFA can treat significantly longer BE with HGD/IMC than EMR alone, with the same efficacy. A multimodal treatment strategy (EMR plus RFA) does not increase the complication rate of endotherapy. EMR is mandatory to ensure a correct histological diagnosis of HGD and IMC	Heterogeneity in treatment results caused by financial problems	Prior EMR or ESD followed by focal RFA (HALO 90) (In a mean number of 1.6 sessions) PLUS additional circumferential RFA (HALO 360) (If needed, one session)
Baldaque‐Silva et al. [32], 2013	Case report 3 (3/0) LGD HGD	Initial experience of gastric RFA in the treatment of gastric dysplasia	RFA might be considered in the treatment of patients with gastric dysplasia	The sample size was small	For LGD: RFA (HALO 90, 15 J/cm^2^, 40 W/cm^2^) For HGD foci with negative deep margin: EMR plus RFA For HGD with negative deep margin and positive lateral margin for LGD: EMR plus RFA
Leung et al. [33], 2015	Clinical trial 12 (9/3) LGD, IM	Using endoscopic radiofrequency ablation to treat gastric dysplasia and metaplasia	RFA successfully eradicated gastric LGD. Gastric IM persisted after RFA, but most patients had evidence of histological improvement on follow‐up.	The sample size was small.	HALO 90 (15 J/cm^2^, 40 W/cm^2^) For 8‐week intervals, maximum of three sessions
Wang et al. [31], 2022	Retrospective 253 (167/86) LGD	Gastric LGD: the effectiveness and prognostic risk factors of RFA	RFA is a safe and effective treatment strategy for gastric LGD. *H. pylori* infection and LGD course > 1 year are major risk factors for relapse.	Single‐center retrospective study Short‐term follow‐up period	HALO 90 (15 J/cm^2^, 57 W/cm^2^) three times for each lesion
Smulian et al. [39], 2014	Safety and tolerability 12 (NR) Anal high grade squamous intraepithelial lesion (HSIL) (HIV‐infected)	Anal HSIL: the safety and tolerability of RFA of the anal mucosa	Safety and tolerability of RFA delivered to the anal mucosa	The use of prescribed analgesia medication after the procedure was not quantified.	HALO 90, HALO flex, (12 J/cm^2^)
Goldstone et al. [4], 2017	Prospective 21 (18/3) Anal HSIL (HIV‐negative)	Anal HSIL: the efficacy and safety of hemi‐circumferential RFA treatment of anal HSIL in HIV‐negative participants	Hemi‐circumferential RFA is an effective and safe treatment modality for managing anal HSIL in HIV‐negative patients.	The sample size was small. The results were from a single center. Participants were consisted of HIV‐negative patients and mainly have a decreased HSIL recurrence rate and low probability to SCC compared to HIV‐positive.	HALO 90, HALO flex, (12 J/cm^2^) every 3 months
Goldstone et al. [40], 2017	Prospective 10 (10/0) Anal HSIL (HIV‐negative and positive)	Anal HSIL: the safety and efficacy of circumferential RFA	Circumferential RFA is fast and safe, and appears to reduce recurrence over targeted ablation and topical treatment of anal canal HSIL.	The sample size was small and encompassed only men patients. The results were from a single center.	HALO flex, HALO 60 (12 J/cm^2^)
Vergara‐Fernandez et al. [41], 2021	Retrospective 12 (7/5) Anal HSIL (immunodeficiency)	Anal HSIL: the efficacy of RFA, and the functional and anatomical changes related to RFA	Focal plus circumferential RFA had a 58.3% efficacy rate in anal HSILs in immunocompromised patients. Patients achieved a CR of the solitary lesions after complementary treatment with electrocautery ablation. RFA halted the development of metachronous lesions.	The sample size was small. The results were from a single center. Collected data in a prospective fashion was retrospectively analyzed, which can be a source of bias. Short term follow up period (mean 18‐month follow‐up)	HALO flex, HALO 60 (12 J/cm^2^) five pulsations at HSILs followed by two overlapping pulsations to cover the entire anal transition zone 3‐months follow up by high‐resolution anoscopy (HRA)
Volante et al. [42], 2009	National survey 3405 (0/3405)	Cervical IEN: the correlation between colposcopic findings and histology	The most commonly used procedure was radiofrequency excision in CIN2‐3. Radiofrequency devices and laser vaporization are usually applied in both large exocervical and endocervical lesions.	Because of the incomplete registration, many of cases with failure in treatment had not been identified.	—
Ronco et al. [43], 2011	National survey 5734 (0/5734)	Cervical IEN: the correlation between colposcopic findings and histology	The most commonly used procedure was radiofrequency excision in CIN2‐3. Radiofrequency devices and laser vaporization are usually applied in both large exocervical and endocervical lesions.	There were discrepancies between colposcopic and histological findings because of the bias in registration.	—
Kornovski et al. [44], 2021	Retrospective 101 (six females treated with RFA) CIN2, CIN3, HGSIL, CIN1 (persistence over 1 year)	Cervical IEN: treatment by RFA compared to another procedure	Successful, safety, and applicability of ablative methods in the outpatient setting over time	RFA and Large Loop Excision of the Transformation Zone (LLETZ) require anesthesia procedure and lidocaine administration.	—

#### 3.3.3 Complications and Adverse Effects of RFA Treatment in Gastric IENs

No evidence of perforation, bleeding, or infection was found after RFA, and the patients did not require antibiotic prophylaxis. The most common complication found was epigastric pain, which can last up to a week and can be easily managed with acetaminophen (Table 1) [31, 32].

### 3.4 Anal IENs

Anal cancer is less common among the general population, and its prevalence is reported to be close to 1.5 per 100,000 [45]. However, high‐risk communities such as human immunodeficiency virus (HIV) patients, homosexual men, positive human papillomavirus (HPV), immunocompromised patients, and transplant recipients on immunosuppressive agents are at higher risk for developing anal cancer [46]. Some studies have reported an incidence of anal cancer in high‐risk individuals of up to 118 per 100,000 [47]. The eradication of the precancerous stage called intracellular neoplasia is the cornerstone of anal cancer prevention. For this purpose, many procedures other than RFA, including topical Trichloroacetic acid, infrared photocoagulation, targeted ablation, electrocautery, and CO2 laser, have promising results in treating IEN. There is a considerable limitation related to the complete excision of the area by electrosurgical excision or cone biopsy because of its high probability of anal stricture and stenosis; hence the role of ablation therapy is highlighted [48, 49]. As the satisfactory findings associated with RFA administration in the treatment of esophageal IEN, studies have tried out radiofrequency energy to clean anal region IEN.

#### 3.4.1 Dosage and Tolerability of RFA in Anal IENs

RFA therapy in anal IENs is suggested by 12.5% of the included studies(Table 1). A study was designed to find the appropriate dose of RFA and its tolerability in 12 patients with anal IEN. They suggested that 3 to 2 pulses were tolerable for the patients, and the anus tissue was well repaired after 4 weeks. Hemi‐circumferential RFA (180°) in 2 or 3 pulses, followed by three rapid RFA sessions after final examinations, was safe and tolerable. This survey facilitates the RFA application for further studies to assess the efficiency, recurrence rate, and potential side effects [39].

#### 3.4.2 Efficacy and Potential of RFA in Anal IENs

The safety and potency of the RFA administration in anal IENs were investigated by Goldstone et al. They followed HIV‐negative patients with high‐grade anal IEN treated with hemi‐circumferential RFA for 1 year. The recurrence rate was approximately 29%, with a 1‐year survival rate of 76% (Table 1) [4]. Further analysis showed that the physicians' skills in using RFA techniques increased simultaneously with the study timeline progression, resulting in significantly lower relapse rates in the last 14 patients than in the first seven patients. HIV‐positive patients could also be managed well by circumferential RFA (with a 90% 1‐year survival rate). Additionally, the risk of metachronous recurrence was less in radiofrequency ablation compared to other ablation methods [40, 41]. Interestingly, combined radiofrequency energy with electrocautery ablation raised the CR rate by 100% in HIV‐positive and immunocompromised patients. In patients with anal IENs, RFA positively impacted sexual function and increased the general health domain measured by Massachusetts General Hospital‐Sexual Functioning Questionnaire (MGH‐SFQ) and Short Form 36 Health Survey Questionnaire (SF‐36) [41].

### 3.5 Cervical IENs

Cervical intraepithelial neoplasia (CIN) is a precancerous condition affecting the epithelium of the cervix transformation zone (TZ). CIN1 is considered a low‐grade squamous intraepithelial lesion (LGSIL) and affects the lower third of epithelium thickness. CIN2 and CIN3 are considered high‐grade squamous intraepithelial lesions (HGSIL) and affect the two‐thirds and entire epithelium thickness, respectively. Cervical cancer is one of the most prevalent malignancies in women and still causes mortality in developing countries. However, a tiny percentage of CIN cases progress to cervical cancer [50, 51]. HPV is the most crucial factor that can lead to CIN. High‐risk HPV (hrHPV), such as HPV‐16 and HPV‐18, are responsible for most cervical cancers. Because of the substantial impact of HPV, routine screening programs should be considered at specific ages, and women with positive results should seek extensive workups and treatment if needed (Table 1) [50, 52].

#### 3.5.1 Ablation Therapy in Cervical IENs

For CIN1 cases, no specific treatment was recommended unless the lesions were visibly present. For CIN2 and CIN3, treatment would be highly regarded and capable by excisional methods (cold knife conisation, loop electrosurgical, and hysterectomy) and ablative methods (thermal ablation, cryotherapy, and CO2 vaporization) [53, 54, 55, 56]. Decision‐making for these patients is usually based on colposcopy findings, equipment, and expertise. Three types of lesions would be determined based on colposcopy findings as completely ectocervical and fully visible (type 1), endocervical and fully visible (type 2), and endocervical part in which the upper border is not fully visible (type 3). Ablative therapy should not be performed in case of the suspicion of invasion or fully visible lesion boundaries. Another contraindication for ablative treatment is the presence of glandular lesions, which should be addressed by excisional therapy. Before the ablation treatment for CIN (like thermal ablation/cold coagulation and cryotherapy), the physician should ensure that the probe tip reaches the border of the lesion [44, 51].

## 4 Conclusion

As a minimally invasive, safe, and efficient procedure, RFA is highly esteemed to address the lesions associated with BE, gastric, anal, and cervical IEN. However, the scope and gaps related to RFA therapy require to be explored precisely.

## Author Contributions

**Hamid Reihani:** conceptualization, software, methodology, data curation, supervision, project administration, validation, visualization, writing – review and editing, writing – original draft, and investigation. **Sepehr Ebrahimi‐Dehkordi:** conceptualization, investigation, writing – original draft, writing – review and editing, project administration, methodology, validation, and data curation. **Reza Moshfeghinia:** conceptualization, investigation, writing – original draft, writing – review and editing, visualization, and data curation. **Amirhossein Ghiasi‐Nejad:** writing – review and editing, writing – original draft, conceptualization, methodology, and data curation. **Kiarash Noorizadeh:** data curation, visualization, validation, methodology, conceptualization, and investigation. **Reza Heidari‐Soureshjani:** conceptualization, investigation, writing – original draft, writing – review and editing, project administration, supervision, resources, data curation, and methodology.

## Conflicts of Interest

The authors declare no conflicts of interest.

## Transparency Statement

The lead author, Hamid Reihani, affirms that this manuscript is an honest, accurate, and transparent account of the study being reported; that no important aspects of the study have been omitted; and that any discrepancies from the study as planned (and, if relevant, registered) have been explained.

## Data Availability

The authors confirm that all data supporting the findings of this study are included in the article. Additional supplementary materials are available upon request from the corresponding author.
